# Review of Integrated Gate Driver Circuits in Active Matrix Thin-Film Transistor Display Panels

**DOI:** 10.3390/mi15070823

**Published:** 2024-06-25

**Authors:** Min-Kyu Chang, Seoyeong Jeong, Darren Kim, Hyoungsik Nam

**Affiliations:** 1 Department of Information Display, Kyung Hee University, Seoul 02447, Republic of Korea; mkchang@khu.ac.kr (M.-K.C.); tjdud0723@khu.ac.kr (S.J.); 2 Harman International, Novi, MI 48377, USA; taesungkim99@gmail.com

**Keywords:** active matrix display, gate driver, shift register, thin-film transistor, low cost, scanning pulse generation

## Abstract

Many advanced technologies have been employed in high-performance active matrix displays, including liquid crystal displays, organic light-emitting diode displays, and micro-light-emitting diode displays. On the other side, there exists a strong demand for cost reduction, and it is one of the low-cost schemes for integrating the driver circuit in a panel based on thin-film transistor technologies. This paper reviews the overall concept, operation principles, and various circuit approaches in shift registers for scanning pulse generation. In addition, it deals with the implementation of additional functionalities in gate drivers to support pixel compensation, multi-line driving, in-cell capacitive touch screen, pixel sensing, and adaptive scanning region control.

## 1. Introduction

Displays are visual apparatuses that transfer visual information to the user by means of images. While liquid crystal displays (LCDs) [1,2,3,4,5,6,7] have dominated the display market from smartphones to televisions, organic light-emitting diode (OLED) displays [8,9,10,11,12,13] have recently become mainstream in smartphones and tablet PCs. Micro-light-emitting diode (mLED) displays [14,15,16,17,18,19] are attracting a lot of attention as a future display technology to realize high luminance and high reliability in addition to various advantages of self-emissive displays like OLED displays. Additionally, the application extends to augmented reality/virtual reality/mixed reality (AR/VR/MR) head-mounted displays (HMDs) [20,21,22,23,24] and automotive displays [25,26,27,28].

A display consists of numerous pixels that are respectively programmed to control their colors and luminances. Because the number of pixels is in the millions or more, it is impossible to update them with a tremendously large number of dedicated lines. Thus, a matrix driving scheme with column and row lines has been employed to reduce the number of lines that would access pixels [29,30,31,32,33,34,35,36,37,38]. Pixels are addressed line by line, where only pixels in a line are programmed at the same time and pixels of other lines are sequentially updated. This line-by-line update operation is called scanning. Usually, column signals carry the pixel voltages to update and row signals control the timing to program the pixels of lines corresponding to column signals. There are two methods of passive matrix (PM) and active matrix (AM) depending on the existence of switching thin-film transistors (TFTs) in pixel circuits. While column lines of PM are directly connected to pixel electrodes, the column lines of AM are linked through switching TFTs, which are controlled by signal levels of row lines. Therefore, pixels of AM can hold the programmed voltage levels during an entire frame time, unlike those of PM holding only for a line time. In matrix driving displays, column signals are provided by source drivers, and row signals are driven by gate drivers. A source driver is also referred to as a data driver or a column driver, while a gate driver is called a scan driver or a row driver.

The key role of gate drivers focused on in this paper is generating scanning pulses to turn on and off switching TFTs in pixels line by line. When the gate drivers are implemented in external complementary metal–oxide–semiconductor (CMOS) integrated circuits, simple shift registers are employed by cascading D flip-flops [39,40,41,42]. Lately, gate driver circuits have been integrated in a display panel to curtail the manufacturing cost [43,44,45,46]. Unlike CMOS circuits with two available types of N-type and P-type MOS transistors, the integration at the TFT backplane should cope with the challenge of implementation with only one-type transistors. TFTs are fundamental components for large-size active-matrix flat panel display products. In the beginning, hydrogenated amorphous silicon (a-Si:H) TFTs were commercialized in LCD panels as switching devices and also used to integrate circuits into a panel [47,48]. However, because a-Si:H TFTs have a mobility lower than 1 ${\mathrm{cm}}^{2}/\mathrm{Vs}$, they have been replaced with low-temperature polycrystalline silicon (LTPS) TFTs with a mobility of more than 100 ${\mathrm{cm}}^{2}/\mathrm{Vs}$ for high-resolution and high-frame-rate displays, especially AMOLED displays requiring high drivability to convert pixel voltages into currents [49,50]. Recently, amorphous oxide semiconductor (OS) TFTs are expanding their applications from large-size to even small-size displays due to their low manufacturing cost and the in-between mobility of a-Si:H and LTPS TFTs [51,52,53,54,55,56]. Besides the generation of scanning pulses, additional compensation and functionalities have been required to be realized in gate drivers.

The contributions of this paper are as follows:

- Providing the most comprehensive review about various technologies of the TFT gate drivers integrated in a display panel;
- Covering the circuit design methodologies of TFT gate drivers at all available TFT backplane technologies;
- Including special circuit implementations in TFT gate drivers for various functions, besides scanning pulse generation;
- Proposing future directions for research on TFT gate drivers.

This paper is organized as follows: Section 2 addresses the overview of the design methodologies of gate drivers integrated at all available TFT backplane technologies, and then Section 3 describes various studies on special circuit implementations of additional functionalities for a variety of display applications. Section 4 concludes this paper with some suggestions on future directions.

## 2. Scanning Pulse Generation Circuits for Integrated TFT Gate Drivers

### 2.1. Basic Structure with N-Type TFTs

**Figure 1:**
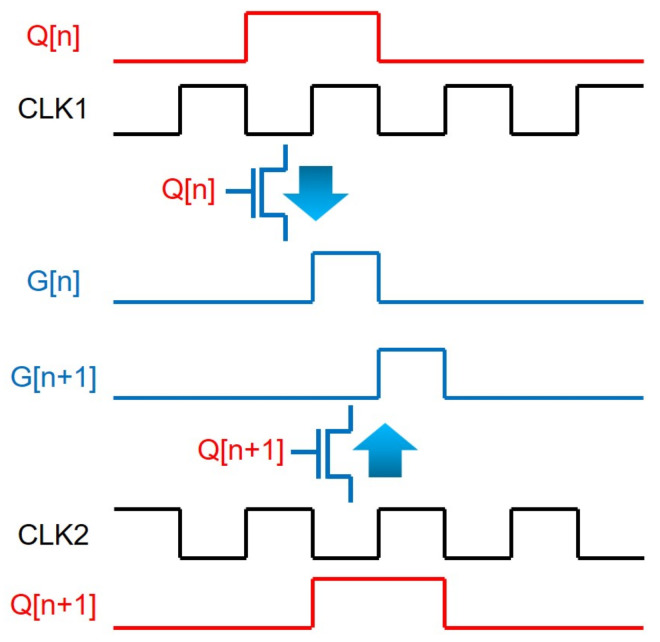
Concept diagram of scanning pulse generation with one-type TFTs.

TFT gate drivers have also been implemented by cascading shift registers, but those TFT shift registers have been realized in a different way from the sampling and holding operations of D flip-flops employed at a CMOS backplane. Because of the availability of only one-type TFTs, the scanning pulses (G[n], G[n + 1]) are generated by sequentially passing one pulse of given two-phase clock signals (CLK1, CLK2) per shift register at the corresponding timing to the current line, as shown in Figure 1, which uses N-type TFTs turned on by high voltages at gate nodes as switching devices. Since the clock repeats high-voltage pulses following low-voltage intervals, one pulse is allowed to be passed by turning on switching TFTs with a high gate-node voltage during the whole period of the clock signal. The overlapped consecutive gate-node pulses (Q[n], Q[n + 1]) of TFT switches produce the scanning pulses of G[n] and G[n + 1].

**Figure 2:**
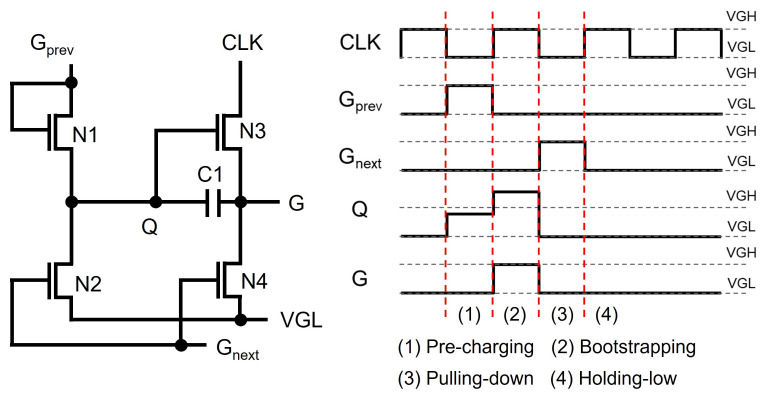
Schematic and timing diagram of a basic shift register at an N-type enhancement-mode TFT backplane.

**Figure 3:**
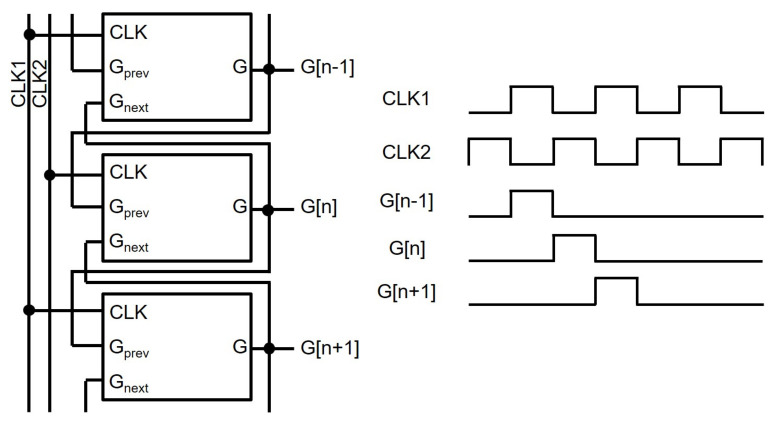
Signal connections between basic shift registers.

The basic structure of a TFT shift register at the backplane of N-type amorphous silicon (a-Si) TFTs [57,58] is depicted in Figure 2, where four TFTs (N1, N2, N3, N4) and one capacitor (C1) are employed with a low supply voltage level of VGL. The operation is explained by three phases of Pre-charging, Bootstrapping, and Pulling-down. In Pre-charging, a previous scanning pulse ($G_{\mathrm{prev}}$) pulls up Q through N1 turning N3 on, while N2 and N4 are turned off. Because CLK stays at the low voltage of VGL, the output (G) stays at the low level. In Bootstrapping, N1 is turned off by the low voltage of the output from a previous stage ($G_{\mathrm{prev}}$) while keeping N3 and N4 turned off. The rising transition of CLK to VGH increases the voltage of G, but there should exist a voltage difference of $V_{\mathrm{TH}3}$ between gate and source nodes of N3. VGH is the highest voltage level used in the gate driver circuit, and $V_{\mathrm{TH}3}$ is a threshold voltage of N3. The lowered output pulse is used to pre-charge a Q-node of the following shift register in which the output voltage level is further reduced, causing the pulse to vanish. To cope with this vanishing issue, C1 is placed between Q and G. Because Q is a floating node at the high-voltage level in the Bootstrapping phase, the voltage across C1 is retained by keeping N3 turned on. This is called a bootstrapping effect. Therefore, CLK is allowed to be transferred to G without any voltage degradation. In Pulling-down, the next scanning pulse of $G_{\mathrm{next}}$ turns on N2 and N4, discharging both Q and G. After this, all TFTs are turned off, maintaining Q and G at low-voltage levels in Holding-low. The signal connections of basic shift registers for generating scanning pulses are described with two out-of-phase clocks (CLK1, CLK2) shown in Figure 3.

### 2.2. Fluctuation Reduction

One issue of the basic structure is having long intervals of low-voltage Q and G nodes at floating states for most of the frame time. Because the drain of N3 is connected to a constantly toggling clock signal, CLK, the coupling noises through parasitic capacitors between drain, gate, and source nodes of N3 cause the fluctuation on Q and G, resulting in undesired pulses.

**Figure 4:**
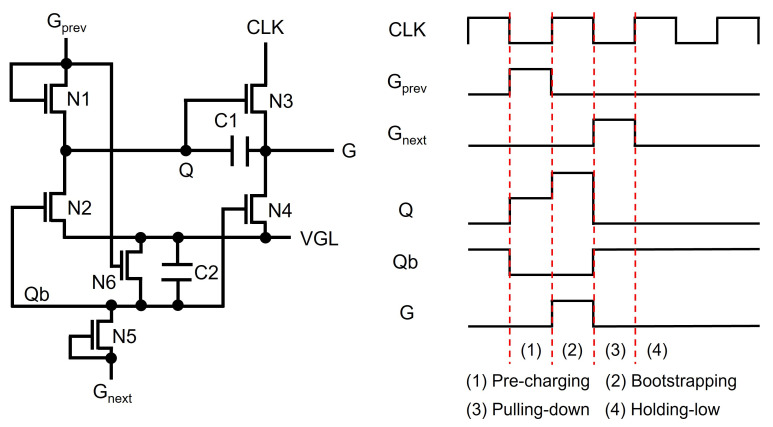
Fluctuation-reduced shift register by applying another high floating node voltage to the gate nodes of pulling-down TFTs at an enhancement-mode TFT backplane.

Most of the recent TFT shift registers keep pulling down Q and G nodes to the low supply voltage instead of maintaining low-voltage floating states. Jang et al. used another floating node to keep turning on switching TFTs of N2 and N4 connected from Q and G to VGL, as illustrated in Figure 4 [59]. Since a Qb-node is maintained at the high-voltage floating state during the whole Holding-low phase, Q and G can stably stay at the low-voltage level through N2 and N4. In addition, the Qb-node is away from CLK, unlike Q and G that are affected through N3; therefore, the fluctuation effect can be substantially reduced.

**Figure 5:**
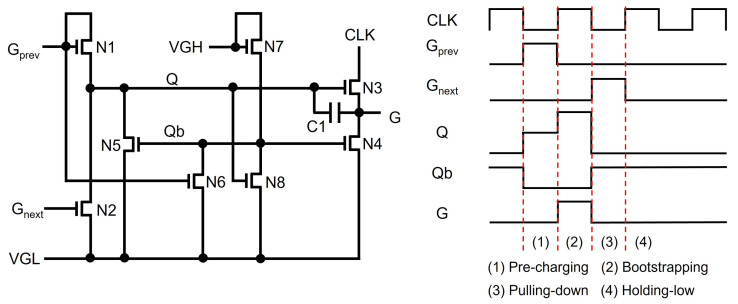
Fluctuation-reduced shift register by including an internal inverter to generate Qb at an enhancement-mode TFT backplane.

As another scheme, an internal TFT inverter [60,61] is included to generate the gate voltage (Qb) of switching TFTs (N4, N5) pulling down Q and G, as shown in Figure 5. While high Q turns off pulling-down TFTs by low Qb, low Q turns them on by high Qb, discharging Q and G nodes. When pre-charging Q, $G_{\mathrm{prev}}$ compels Q to become high, leading to low Qb. When pulling down Q, $G_{\mathrm{next}}$ forces Q to be low, leading to high Qb. However, as the internal inverter is implemented by discharging TFT (N8) and diode-connected load (N7) that are connected to VGL and VGH, respectively, the high Q-node turns both TFTs on, generating a shoot-through current path along with a high power consumption.

**Figure 6:**
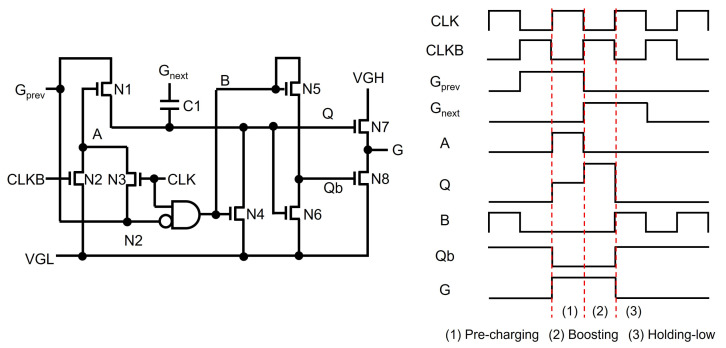
Low power shift register by removing the shoot-through current path in an internal inverter at an enhancement-mode TFT backplane [62].

As to this shoot-through current issue, it has been proposed to link the diode-connected load to a clock signal or a toggling internal node voltage instead of the constant VGH. As presented in Figure 6 [62], where the scanning pulse width is twice as that of one line time, because a B node is pulled down for the period of high Q including pre-charging and boosting phases, and is connected to the diode-connected load, any shoot-through current cases have not existed in the internal inverter of N5 and N6, leading to the substantially reduced power consumption.

### 2.3. Reliability Enhancement over $V_{TH}$ Shifts

**Figure 7:**
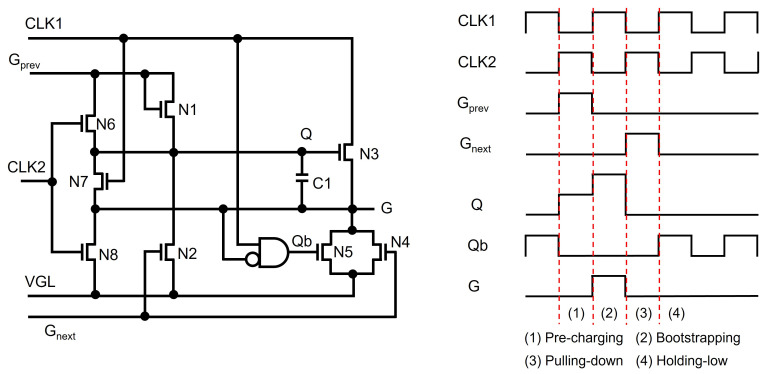
Reliability-enhanced shift register by the line-by-line alternation of discharging paths for Q and G at an enhancement-mode TFT backplane [67].

One of the important problems of a-Si and oxide TFTs is the $V_{\mathrm{TH}}$ shift caused by the gate voltage stress, leading to a limited operational lifetime [63,64]. Especially, pulling-down TFTs for Q and G nodes severely experience the $V_{\mathrm{TH}}$ shift because their gate nodes are driven at high-voltage levels for most of the frame time. This has been addressed by applying two pulling-down TFTs to one node and then using them alternately in a line-by-line [65,66,67,68] or frame-by-frame fashion [69,70]. S. H. Moon et al. used two TFTs (N5, N8) alternately at every line time, as presented in Figure 7 [67]. In addition to the basic structure of N1 to N4, in Holding-low, Q is discharged through N5 and N7 to VGL during high CLK1 and through N6 to low $G_{\mathrm{prev}}$ during low CLK1. G is pulled down via N5 during high CLK1 and via N8 during low CLK1. Therefore, while the pulling-down paths for Q and G are secured, the lower stresses on pulling-down TFTs contribute to the reduced $V_{\mathrm{TH}}$ shift.

**Figure 8:**
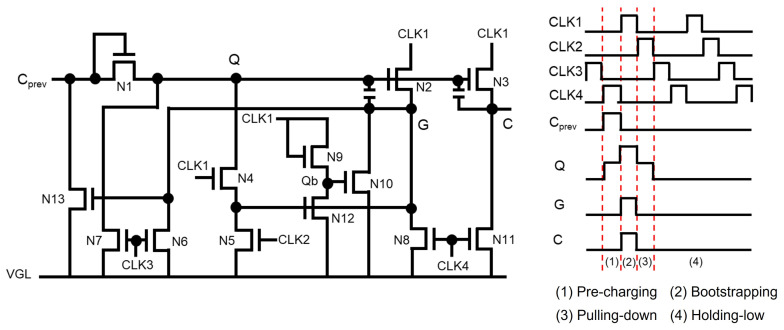
Four-phase clock shift register for reliability enhancement and power reduction at an enhancement-mode TFT backplane [72].

As described in Figure 8, there exist shift registers with three or more clocks of smaller duty ratios and lower frequencies to achieve higher reliability and lower power consumption [71,72,73]. Because more pulling-down TFTs are used to pull down Q and G nodes with relatively lower stress, the overall reliability is improved with the increased number of TFTs. G is stably linked to VGL during the Holding-low period with four TFTs of N5, N6, N8, and N10 controlled by four clocks of CLK1, CLK2, CLK3, and CLK4. In addition, the smaller capacitive load and lower operating frequency of clock signals achieve the reduction on power consumption.

**Figure 9:**
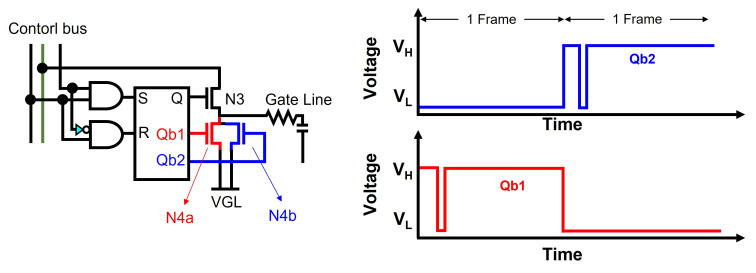
Reliability-enhanced shift register by the frame-by-frame alternation of discharging paths for Q and G at an enhancement-mode TFT backplane.

The frame-by-frame control of these pulling-down TFTs [69,70] has also been proposed to improve the reliability, as illustrated in Figure 9. Two different Qb1 and Qb2 nodes are applied to gate nodes of two pulling-down TFTs (N4a, N4b), respectively, while they are asserted in turns at every frame time. Therefore, N4a is turned on for odd frames and N4b is turned on for even frames, leading to a smaller $V_{\mathrm{TH}}$ shift.

### 2.4. P-Type TFT Circuit Implementation

Unlike N-type a-Si TFTs, LTPS TFT backplanes have mainly used P-type TFTs for manufacturing AM displays [74,75,76,77,78,79]. Because P-type TFTs work opposite to N-type TFTs, we have to change the point of view for the operation of shift registers. Since the low level pulses at gate nodes are considered as signals to turn on TFTs, the pulling-down is the pulse generation and the pulling-up means resetting the shift register for the rest of the frame time. Bootstrapping effects also take place in a falling direction to guarantee that the pulling-down TFTs are sufficiently turned on without any output voltage losses at low-voltage levels.

**Figure 10:**
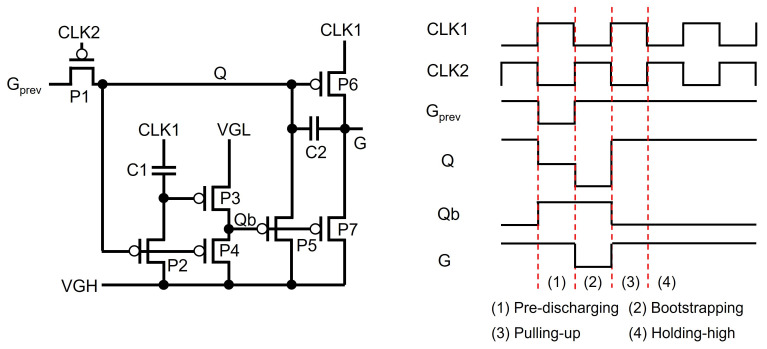
Shift register based on P-type enhancement-mode TFTs [78].

As presented in Figure 10 [78], in Pre-discharging, Q is discharged through P1 by the low pulses of $G_{\mathrm{prev}}$ and CLK2. This causes G to be high voltage with P6 by CLK1, pulling up Qb to turn off P5 and P7. In Bootstrapping, the Q-node at the floating low-voltage level goes down to the more negative voltage level by the capacitive coupling of C2 from G to Q with the falling transition of CLK1. Therefore, G can reach exactly the low level of CLK1 without any degradation. In Pulling-up, Q is charged through P1 by $G_{\mathrm{prev}}$, turning off P2, P4, and P6. Thus, the internal inverter composed of P2, P3, P4, and C1 pulls down Qb to VGL, charging Q and G up to VGH through P5 and P7, respectively. In Holding-high, Qb stays at VGL to keep Q and G at the VGH level through P5 and P7.

### 2.5. Depletion-Mode TFT Circuit Implementation

**Figure 11:**
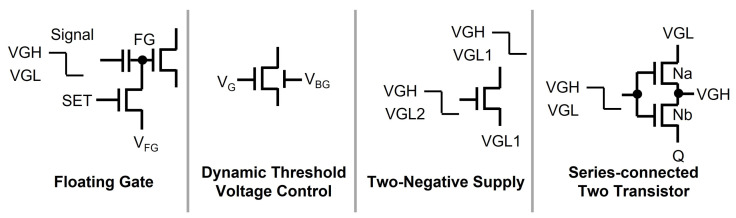
Approaches to deal with depletion-mode TFTs.

These days, oxide TFTs have become the mainstream of TFT backplane technologies due to their high mobility and low manufacturing cost; however, they have some challenges to take into account during the circuit design. One is the fact that N-type oxide TFTs are fabricated with negative $V_{\mathrm{TH}}$; that is, they are depletion-mode transistors where TFTs are turned on even at zero $V_{\mathrm{GS}}$. Some methods have been reported to cope with this depletion-mode issue in TFT shift registers such as floating gate, dynamic threshold voltage control, two-negative-supply, and series-connected two-transistor (STT) schemes [80,81,82,83,84,85,86], as explained in Figure 11.

The floating gate scheme connects the internal signal via the capacitor to the gate node (FG). Because the FG node is programmed with $V_{\mathrm{FG}}$ at the signal of VGH and the voltage across the capacitor is set to VGH $-V_{\mathrm{FG}}$, the low-voltage level of FG can go down to that lower than VGL by VGH $-V_{\mathrm{FG}}$, ensuring that a TFT is turned off due to the negative $V_{\mathrm{GS}}$. The dynamic threshold voltage control adjusts the threshold voltage of a dual-gate TFT to become positive by applying the control voltage to the bottom gate ($V_{\mathrm{BG}}$); that is, the depletion-mode TFT changes into the enhancement-mode one. Therefore, it is guaranteed that the zero $V_{\mathrm{GS}}$ turns off oxide TFTs. In the two-negative-supply, the gate node voltage is generated with the lower voltage level of VGL2 than VGL1 used in source and drain nodes of TFTs, guaranteeing that a TFT can be shut off by the negative $V_{\mathrm{GS}}$. STT uses two transistors (Na, Nb) connected in series. In the bootstrapping phase that needs to maintain Q at the high-voltage floating state, Nb can be perfectly turned off by asserting its source and drain nodes with high voltages, that is, the negative $V_{\mathrm{GS}}$.

**Figure 12:**
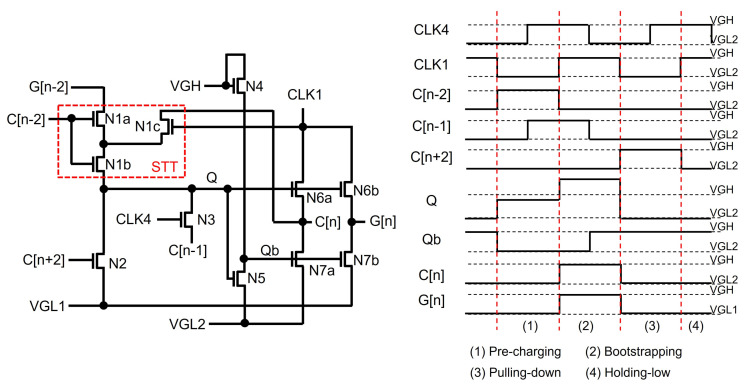
Shift register based on N-type depletion-mode oxide TFTs [82].

Among them, two-negative-supply and STT schemes are widely deployed to address the depletion-mode issue. The shift register in Figure 12 [82] has two negative supplies of VGL1 and VGL2, where VGL2 is more negative than VGL1. Because the sizes of N6b and N7b should be large enough to drive heavy capacitive and resistive line loads by the output pulse (G[n]), it is of great importance to be able to perfectly turn off N7b without the shoot-through current path. Therefore, Qb is generated by the internal inverter of N4 and N5 supplied with VGH and VGL2, allowing N7b to be shut off because its $V_{\mathrm{GS}}$ can be further negative than $V_{\mathrm{TH}}$. This circuit also produces another output pulse (C[n]) with two voltage levels of VGH and VGL2, while G[n] has voltage levels of VGH and VGL1. Consequently, Q can be maintained at the floating state by turning off N1a, N1b, and N2 when C[n−2] and C[n+2] are lower with VGL2 than VGL1. Especially, an STT scheme is employed with N1a, N1b, and N1c in the red box. N1b’s source is asserted by the high-voltage Q, and its drain is connected to the high-voltage C[n] through N1c in the Bootstrapping phase. Therefore, the C[n−2] of VGL2 sets N1b to be cut off with the negative $V_{\mathrm{GS}}$.

### 2.6. Node-Sharing Schemes for Small Area Implementation

**Figure 13:**
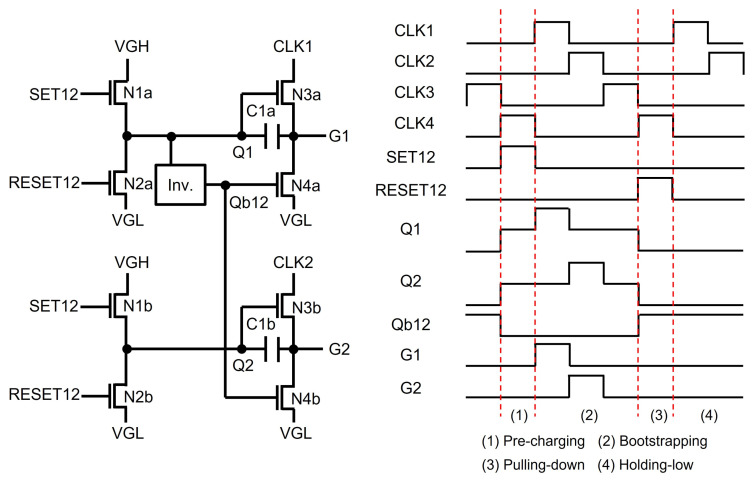
Small area shift register with a Qb-sharing structure at an enhancement-mode TFT backplane [87].

There have been studies on reducing the area of TFT gate driver circuits for narrow bezel panels. This area reduction has been achieved by employing a smaller number of TFTs in a shift register with the node sharing schemes of Qb and Q-nodes over multiple stages. Qb has been shared by holding it at a low voltage for multiple lines while extending the floating state Q for the same period of time. Because the floating Q-node must be bootstrapped only once for multiple lines, it requires duty ratio clocks smaller than 50%, increasing the number of clock signals to support the line-by-line scanning. A single Qb generation circuit is employed for multiple lines; therefore, the number of TFTs is reduced. The Qb-sharing architecture, which covers two shift registers, is illustrated in Figure 13 [87]. The same input and reset signals of SET12 and RESET12 are applied to two consecutive shift registers, conducting simultaneous pre-charging and pulling-down operations. Therefore, the high interval of Q1 is equal to that of Q2, and the shared Qb12 is generated by the internal inverter with respect to Q1. To secure enough bootstrapping interval and to enable the separate bootstrapping on Q1 and Q2, four-phase clock signals (CLK1, CLK2, CLK3, CLK4) of 25% duty ratio are employed and four line times are assigned as the interval of low Qb12, including pre-charging and bootstrapping phases of Q1 and Q2.

**Figure 14:**
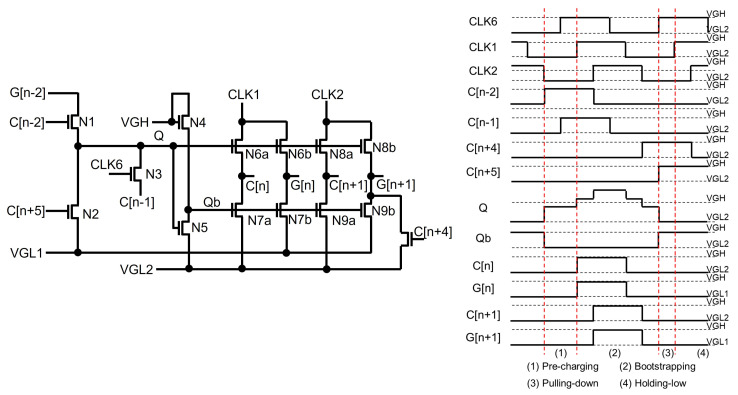
Small area shift register with a direct Q-sharing structure at a depletion-mode TFT backplane [88].

**Figure 15:**
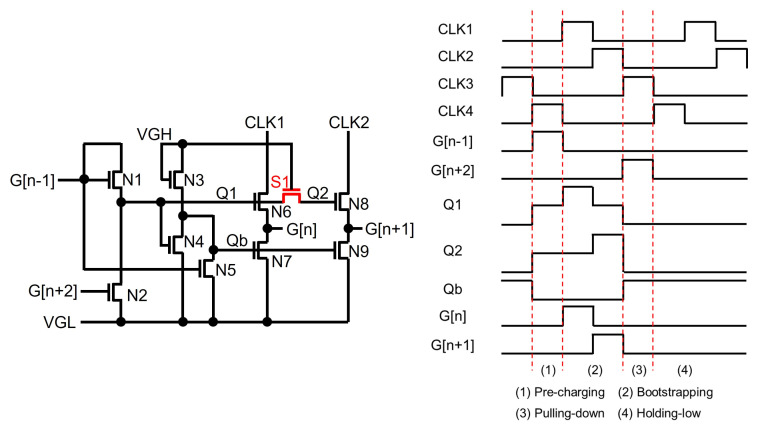
Small area shift register with a Q-sharing structure by separating TFTs at an enhancement-mode TFT backplane [90].

On top of Qb, Q can also be shared by directly connecting it to gate nodes of multiple pulling-up TFTs. However, the bootstrapping effect on Q is weakened because the capacitive coupling effect through one pulling-up TFT is divided by the capacitance of other pulling-up TFTs, as depicted in Figure 14 [88,89] based on oxide TFTs. As a consequence, Q is bootstrapped at the rising transition of CLK1 by the smaller level compared with that without Q-sharing. After the following rising transition of CLK2, the final boosted voltage level of Q is achieved. If there exist no overlaps between CLK1 and CLK2, the bootstrapping level without Q-sharing is not achievable by this simple Q-sharing architecture. To cope with this bootstrapping loss, a separating TFT (ST1) is placed between pulling-up TFTs of N6 and N8, as shown in Figure 15, where the shift register is designed at the backplane of N-type LTPS TFTs [90,91,92]. Because separating TFTs between pulling-up TFTs are turned off during the bootstrapping phase, the gate nodes (Q1, Q2) of pulling-up TFTs are fully boosted without any bootstrapping degradation caused by other pulling-up TFTs.

## 3. Special Gate Driver Circuits

### 3.1. Emission Pulse (EM) Generation

**Figure 16:**
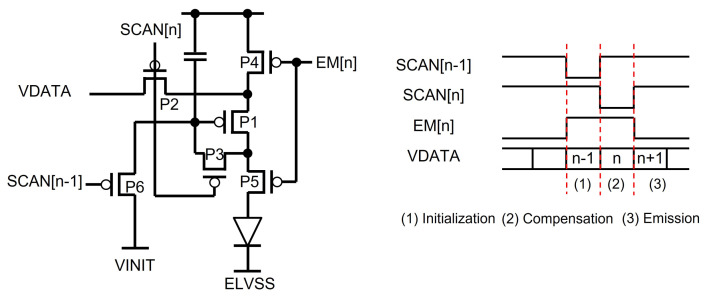
Threshold voltage compensation pixel circuit example [104].

In OLED displays, pixel circuits typically control the brightness by modulating the current supplied to the OLED [93,94]. The driving TFT is responsible for converting the voltage data into currents, ensuring consistent brightness across all pixels on the display panel. However, due to inevitable TFT process variations such as threshold voltage and mobility, it is impossible to guarantee that all driving TFTs provide identical currents for the same pixel voltage data. Therefore, compensation techniques must be implemented in pixel circuits to address these variations [95,96,97,98]. The emission pulse generation circuit is a specialized circuit for generating signals that drive the emission TFTs (EM TFTs) of a pixel circuit for controlling the current flows through OLEDs [99,100,101,102,103]. In general, the threshold voltage compensation is achieved by sensing the threshold voltage of a driving TFT based on the diode connection condition, where EM TFTs cut off the current paths through OLEDs to avoid the undesired light emission and to sense the accurate threshold voltage as presented in Figure 16 [104], where P1 is a driving TFT and P4 and P5 are EM TFTs. Additionally, EM TFTs can be utilized to adjust the emission time, thereby controlling the resultant brightness.

**Figure 17:**
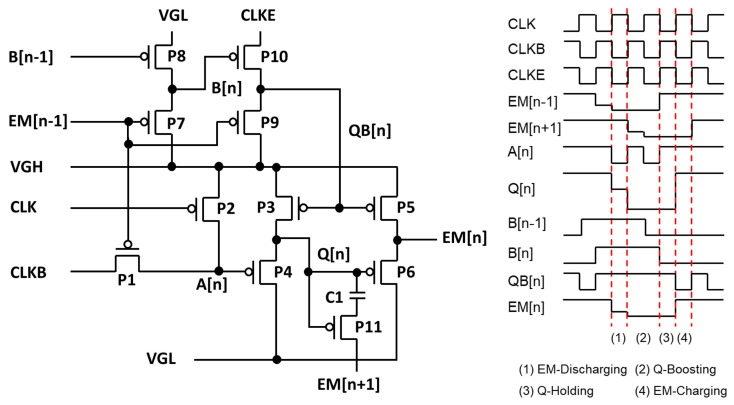
Emission pulse generation circuit based on P-type enhancement-mode TFTs [103].

Thus, the operation of the EM pulse generation circuit should incorporate signal shifting and adjustable longer pulse width instead of scanning pulse width. An example EM pulse circuit is illustrated in Figure 17, where the output signal (EM[n]) is shifted in synchronization with clock signals (CLK, CLKB, CLKE), and its pulse width can be programmed via the pulse width of an input signal or a previous stage’s output (EM[n − 1]) [103]. The circuit is based on a P-type TFT backplane and composed of 11 TFTs and one capacitor, enabling the shift operation and the programmable pulse width. CLK, CLKB, and CLKE are clock signals of a 50% duty cycle, and VGH and VGL serve as the high and low power supplies, respectively. The timing diagram is divided into the four phases EM-Discharging, Q-Boosting, Q-Holding, and EM-Charging. During the EM-Discharging phase, low EM[n − 1] and the falling transition of CLKB cause A[n] and Q[n] to be pulled down to a low-voltage level with one line delay relative to EM[n − 1]. P6 is turned on due to low Q[n], resulting in low EM[n]. Meanwhile, P9 sets QB[n] to VGH by low EM[n − 1], turning off P3 and P5. During the Q-Booting phase, Q[n] is boosted below VGL in the negative direction by the falling transition of the next stage’s EM[n + 1] signal, ensuring the stable low voltage of VGL at EM[n]. In the Q-Holding phase, Q[n] and EM[n] are maintained at a low-voltage level. During the EM-Charging phase, the falling transition of CLKE sets QB[n] to the low voltage, pulling Q[n] and EM[n] up to VGH through P3 and P5, respectively. Consequently, variations on the width of EM[n − 1] result in corresponding changes in the width of EM[n].

### 3.2. Multi-Line Driving

Because virtual reality displays employ lenses between eyes and displays to magnify the screen, very-high-resolution and large-field-of-view (FoV) panels, such as those that are more than 3000 pixels per inch (ppi) and wider than 100°, are required [105,106], enabling a vertical resolution higher than 9000. Additionally, frame rate displays higher than 90 Hz are also demanded to reduce motion-to-photon latency [107]. These requirements cause an insufficient charging time problem regarding driving display panels due to reduced line times.

**Figure 18:**
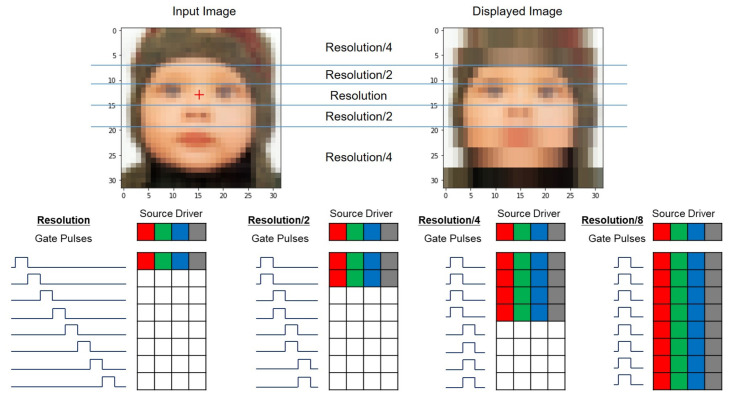
Foveation-based driving schemes with the vertical resolution reduction [108].

To address this issue, the foveation-based driving scheme has been proposed to reduce the effective number of lines in a frame by reducing the vertical image resolution of distant regions from the fixation point [108,109]. It is the high-resolution display driving technique that directly realizes the foveated-rendering images in a panel, as presented in Figure 18. The low-resolution areas are driven by multi-line driving shift registers to be able to provide synchronized pulses to multiple lines at the same time. For 1/2, 1/4, and 1/8 resolution areas, 2, 4, and 8 lines are driven at the same time, respectively, resulting in the reduction in the effective number of lines by the same factors. The multi-line driving shift registers are implemented based on the Q-node sharing architecture described in Section 2.6 [90,91], and the output pulse sequences are adjusted by changing the timing of the clock signals [110]. Effective numbers of lines are reduced to 30.3% and 21.0% for 4800 × 4800 and 9600 × 9600 resolutions, respectively. Consequently, the charging time of a line can be extended to 330.0% and 476.2% for those resolutions.

### 3.3. Robustness Improvement over Noises in In-Cell Touch Applications

**Figure 19:**
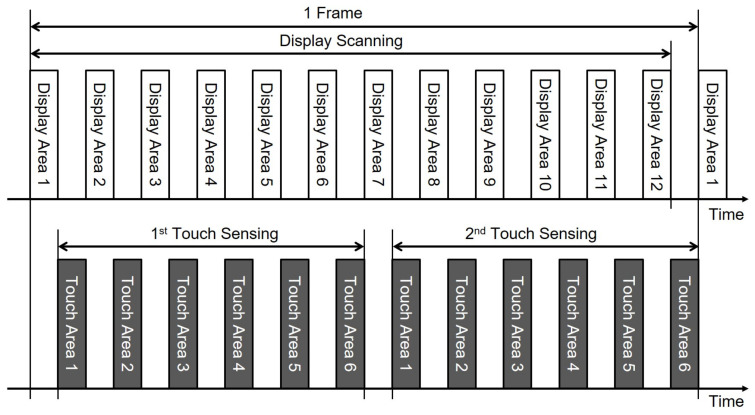
Timing diagram of display scanning and touch sensing at TDDM [112].

Because the capacitive touch screens use capacitive coupling effects to detect touches, the in-cell touch circuit’s performance is also sensitive to the coupling noises from the display circuits such as gate drivers and source drivers. To avoid the degradation of the detection performance by display noises, the touch sensing operation was conducted only during the vertical blank [111], in that no scanning functions worked. Touch sensing only in the vertical blank showed the un-natural representations of moving touch actions, and there was a difficulty in achieving a touch reporting rate higher than the display frame rate. Therefore, the touch sensing intervals have been inserted in the middle of the display scanning operation, as depicted in Figure 19 [112,113,114,115,116]. It is called a time division driving method (TDDM).

**Figure 20:**
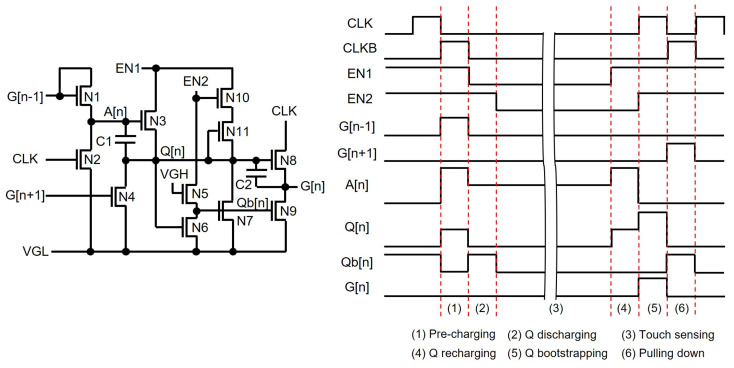
Shift register schematic and timing diagram at an enhancement-mode TFT backplane for a TDDM touch sensing scheme [115].

Thus, the shift registers that can support TDDM should be able to start the pulse generation again after stopping during the touch sensing intervals, as presented in Figure 20, to get rid of display noises at the touch detection. The display noises are controlled during the touch sensing operation by shutting down clock pulses. Low-voltage EN1 and EN2 signals indicate the touch sensing interval, and A[n] stores the information about the pre-charging at the n-th line right before the touch sensing period starts. A[n] is pre-charged through N1 by the previous output (G[n − 1]), and then Q[n] is pre-charged via N3 when EN1 is high. Meanwhile, when EN1 is low during the touch sensing period, Q[n] is discharged to VGL, but A[n] maintains its pre-charged voltage at C1. After the touch sensing interval ends, high EN1 triggers the pre-charging at Q[n] by the high voltage stored at A[n], and then the bootstrapping takes place at Q[n] by the rising transition of CLK, generating G[n] without any voltage losses.

### 3.4. Random Sensing Pulse Generation for External Compensation in OLED Displays

**Figure 21:**
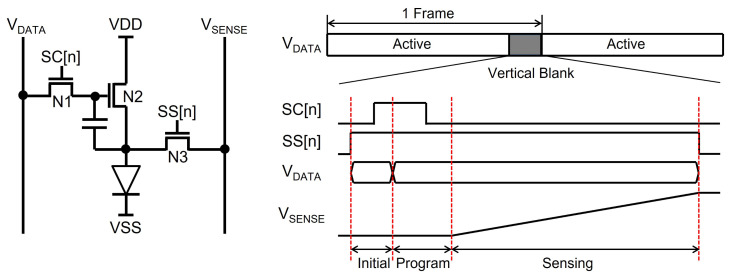
Pixel circuit with a sensing TFT (N3) for the external compensation [125].

**Figure 22:**
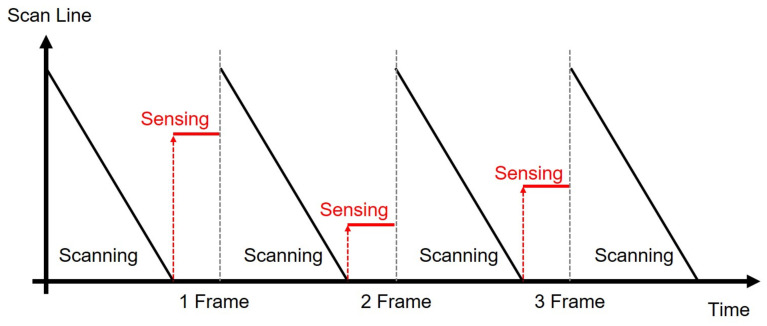
Random sensing line selection during the vertical blank period [125].

While LCDs adjust the pixel luminance by voltage, OLED displays control it by current. Because the source drivers provide the pixel data with the voltage signals, pixel circuits in OLED displays contain driving TFTs to convert pixel voltages into currents. Therefore, the variation on the characteristics of driving TFTs causes visible artifacts on the screen. In addition, the degradation of the OLED efficiency is also perceived as an image sticking phenomenon. To compensate for these artifacts, internal and external compensation methods have been developed. While the internal compensation is categorized into voltage programming and current programming for the compensation of the TFT variation [117,118,119,120,121], the external compensation scheme can address TFT variations as well as OLED degradation to meet the requirement of the long lifetime in large display applications. The pixel circuits based on external compensation methods includes a sensing TFT (N3) to sense the characteristics of TFTs (N2) and OLEDs, as described in Figure 21 [122,123,124]. After the full scanning for a frame of the image is finished, the sensing operation for pixels of one line is conducted in the vertical blank to secure enough sensing time. The sensing operation gets to cover the whole pixel data by selecting another line in a frame-by-frame way. The control signals (SENSE) to turn on sensing TFTs are generated by the additional circuits in gate drivers, and the line selection should be able to be randomly fulfilled in order to avoid perceivable artifacts, as shown in Figure 22 [125,126].

**Figure 23:**
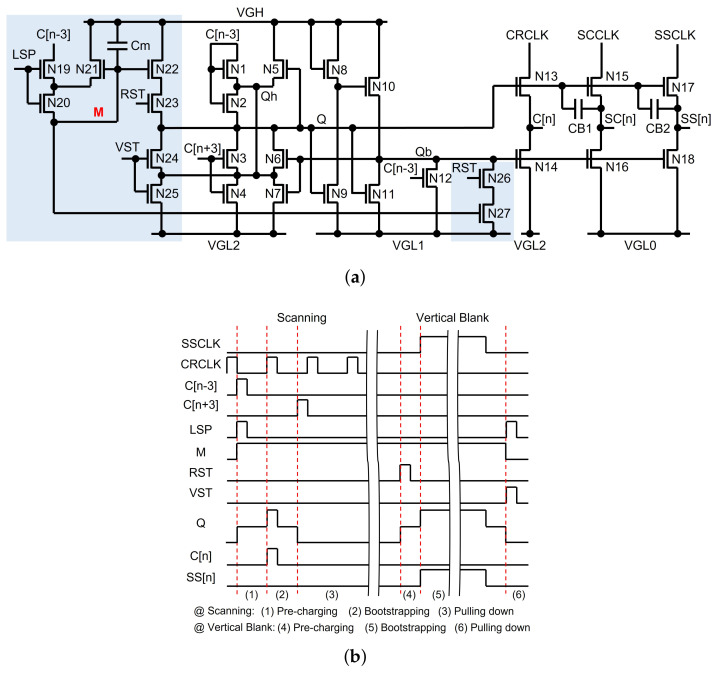
Random sensing pulse generation at a depletion-mode oxide TFT backplane: (**a**) schematic; (**b**) timing diagram [125].

An example circuit supporting the random sensing pulse generation is illustrated in Figure 23a [125]. The timing diagram is also described in Figure 23b, where an n-th line is selected to store the high M-node voltage during the scanning operation and to generate its sensing pulse (SS[n]) during the vertical blank. When LSP is asserted at the high pulse of C[n − 3] in the scanning period, the M-node is charged to the high voltage that is held in the capacitor, Cm. In the vertical blank, while the high pulse of RST causes the pre-charging on the Q-node through N22 and N23, it also pulls down the Qb-node through N26 and N27. Then, the high pulse of SSCLK is transferred to SS[n] without the voltage loss by bootstrapping. Both following high pulses of RST and VST discharge M and Q, pulling Qb up. Because LSP pulses are generated randomly by a timing controller, the random sensing pulse generation is accomplished.

### 3.5. Adaptive Scanning Region Control

The most common method for reducing the power consumption of digital systems is to lower the operating frequency. In display applications, the operating frequency, that is, the refresh rate, causes the trade-off between power consumption and moving picture quality. Whereas the higher frequency improves the picture quality, the lower frequency reduces the power consumption. To tackle this trade-off, the adaptive control schemes have been employed to adjust the display refresh rate depending on the moving image characteristics. These schemes, which are called a variable refresh rate (VRR), increase the refresh rate for fast-moving images and decrease it for slow-moving or still images [127,128,129,130,131]. Usually, VRR displays are implemented by changing the vertical blank length with the fixed scanning time of the active pixel area during the target frame time.

On the other hand, there exist cases that have moving image regions and still image regions at the same time in a screen. Thus, some adaptive scanning region control methods have been proposed with selective scanning shift registers to enable different refresh rates in different regions [132,133,134]. These circuits can block or enable the gate pulse generation at specific regions of specific frames, leading to the different refresh rates over some regions of a display.

**Figure 24:**
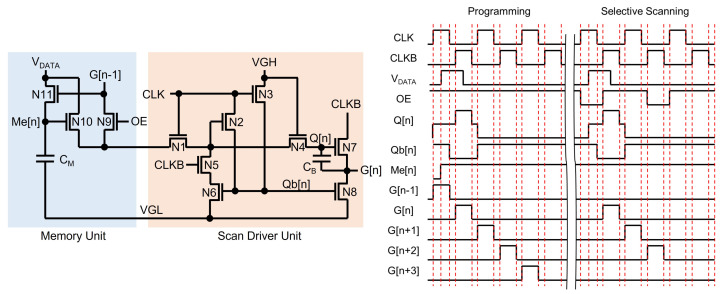
Selective scan driver circuit at an enhancement-mode TFT backplane to enable the gate pulse generation only for the specific region [132].

One selective scan driver is illustrated in Figure 24, which consists of a memory unit and a scan driver unit. The scan driver unit is a shift register, where Q[n] can be pre-charged by G[n − 1] and $V_{\mathrm{DATA}}$ through N9 and N10, respectively. Since the Programming period maintains OE at the high level, Q[n] is pre-charged by G[n − 1], resulting in the generation of all scanning pulses. When the high pulse of $V_{\mathrm{DATA}}$ is applied at an (n − 1)-th line timing of the high G[n − 1] pulse, a Me[n]-node is charged to the high voltage held at Cm, turning on N10 in the n-th circuit. Since the following frame does not apply a start pulse to the first stage, the scanning pulses do not take place. However, when the high pulse of $V_{\mathrm{DATA}}$ and the low pulse of OE are applied, the n-th shift register with the high-voltage Me[n]-node pre-charges Q[n] and generates the high pulse of G[n]. Consequently, the following stages give rise to consecutive pulses until another low pulse of OE is applied. Since this low OE pulse blocks the input signal (G[n − 1]) from the previous shift register by turning off N9, output pulses are not produced anymore. Therefore, the specific pulse generation region is programmed, and the remaining region is displayed by the pixel data updated in the previous frame without scanning pulses.

## 4. Conclusions and Future Directions

This paper describes a variety of technologies for the TFT shift register that is a key circuit block of the gate driver circuit integrated in display panels to reduce the manufacturing cost. Those technologies cover TFT backplanes, reliability improvement, depletion-mode TFT compensation, circuit area reduction, and low power consumption. In addition, it deals with the circuit implementations included in the gate driver for other functionalities, such as emission pulse generation, multi-line driving scheme, noise reduction on touch sensing, random sensing pulse generation, and adaptive scanning region control.

Many studies have continued to improve these kinds of technologies further towards longer reliability, smaller circuit area, lower power consumption, and new TFT backplanes. Recently, the low-temperature poly-crystalline oxide (LTPO) TFT backplane has been developed to leverage the low leakage current of oxide TFTs and the high mobility of LTPS TFTs at the same time [135,136,137,138]. Some papers about the simple and high performance shift registers implemented with LTPO TFTs can be found in well-known circuit-related journals [139,140,141,142].

On top of the main function of displaying images on the screen, more efforts have made good progress in integrating additional functions in display panels, such as image sensing [143,144,145], fingerprint sensing [146,147,148,149,150,151], temperature sensing [152,153,154,155,156], visible light communication [157,158,159,160], and actuators for haptic applications [161]. To support these diverse functions and the display scanning, the demands for higher flexibility in gate driver circuits are going to increase.

These continuous advancements of devices and circuits will pave the bright way to the future of displays.

## Data Availability

Data sharing is not applicable.
